# Gastrointestinal disease in Sjogren’s syndrome: related to food hypersensitivities

**DOI:** 10.1186/s40064-015-1557-7

**Published:** 2015-12-12

**Authors:** Christine Kim-Lee, Lakshmanan Suresh, Julian L. Ambrus

**Affiliations:** Department of Medicine, SUNY at Buffalo School of Medicine, Buffalo, 14203 NY USA; Department of Oral Diagnostic Sciences, SUNY at Buffalo School of Dental Medicine, Buffalo, 14214 NY USA; Immco Diagnostics, 640 Ellicott Street, Buffalo, 14203 NY USA

## Abstract

Patients with Sjogren’s syndrome (SS) frequently have irritable bowel like symptoms (IBS). Some have celiac sprue. The current studies were designed to examine the presence of food hypersensitivities in a population of patients with SS and IBS. Ten patients were selected from the autoimmune disease clinics at SUNY at Buffalo who had SS and IBS symptoms. Food hypersensitivities were determined by specific IgG ImmunoCAP^®^ assays. Symptoms of abdominal pain, bloating, diarrhea and joint pain were eliminated with dietary restriction of foods to which hypersensitivity was demonstrated. Symptoms recurred with re-institution of offending foods. Resolution of fatigue required elimination of offending foods as well as treatment of underlying metabolic disorders. The presence of IBS in patients with SS should lead to investigation of food hypersensitivities as possible culprits.

## Background

Sjogren’s syndrome (SS) is a common autoimmune disease characterized by destruction of the salivary and lacrimal glands leading to symptoms of dry eyes and dry mouth. Extra-glandular manifestations commonly include lung disease, kidney disease and lymphoma (Delaleu et al. 2005; Mavragani and Moutsopoulos 2014). Lymphomas may occur in the salivary glands but also frequently in the gastrointestinal tract (Liang et al. 2014; Mariette 1999). Gastrointestinal disease in SS may occur in one quarter of the patients and include dysphagia, gastritis, motility disorders, pancreatitis, pancreatic insufficiency, pernicious anemia, autoimmune hepatitis and symptoms consistent with irritable bowel syndrome (IBS), abdominal pain, diarrhea, constipation, bloating, flatulence, vomiting and nausea (Ebert 2012; Bengtsson et al. 2011). Some patients with SS and IBS are found to have celiac sprue (Conti et al. 2015). The current studies were undertaken to evaluate if patients with SS and IBS symptoms in our clinic population had hypersensitivities to particular foods, whether or not they met criteria for celiac sprue, and if elimination of the offending foods would alleviate their symptoms.

## Methods

Ten patients were identified from the adult autoimmune disease clinics at SUNY at Buffalo School of Medicine who met the 2012 ACR criteria for the diagnosis of SS and complained of abdominal pain, bloating, diarrhea and fatigue associated with the ingestions of particular foods. All had been diagnosed with IBS or celiac sprue by gastroenterologists. In some cases, patients had identified particular foods as culprits, but in most cases not. The presence of fatigue and joint pain were determined by questioning the patients.

Determination of food hypersensitivities was performed using the ImmunoCAP^®^ system (Phadia AB, Uppsala, Sweden) to measure food-specific IgG. The food was immobilized on the solid phase and IgG antibodies were detected with a fluorescently labeled antibody. The latter detected all subclasses of IgG. The IgG scoring system was established by the manufacturer (Viracor-IBT Laboratories, Lee’s Summit, MO) with a standard curve used to calculate the specific IgG concentrations. The calibrators were referenced to the International reference preparation for serum Ig. The assay range has a lower limit of quantitation of 2 μg/mL and an upper limit of quantitation of 200 μg/mL for specific IgG. The assay was reported as positive when food-specific IgG levels were higher than 2 mcg/mL (Atkinson et al. 2004).

In each case, patients met with a dietician and developed a diet eliminating the foods identified by these assays. At the end of a 6 months period, patients attempted to reinstitute foods, one at a time. Symptoms were recorded before and during the elimination diet as well as with the reinstitution of the culprit foods. These studies were approved by the IRB, SUNY at Buffalo School of Medicine.

## Results

The characteristics of the ten SS patients studied are shown in Table 1. This group consisted of 8 females and 2 males, age 28–63 years (mean 48.2 years). Most patients had SS for 2 years or less, although one patient had disease for 3 years, two for 5 years and one for 18 years. The symptoms of bloating, diarrhea, joint pain and fatigue often preceded the diagnosis of SS. Interestingly, although perhaps because of the nature of the autoimmune disease clinics at SUNY at Buffalo School of Medicine that have a large number of patients with metabolic muscle diseases, all of the patients were identified to have metabolic myopathies. Five patients had carnitine palmitoyl transferase deficiency. One patient had complex 2 disorder of the mitochondrial respiratory chain, and another had lactate dehydrogenase deficiency. All of these patient had their diagnosis confirmed based on muscle biopsies. Three additional patients had their mitochondrial dysfunction demonstrated by elevated lactic acid at rest on several occasions, without an alternative explanation. Besides SS and metabolic disorders, two patients had psoriasis and three had anti-phospholipid antibody syndrome.

**Table 1:** The Table summarizes demographic data in ten patients studied with SS and IBS–like symptoms

Patient	Age (years)	Sex	Duration Sjogren’s syndrome (years)	Other medical comorbidities
1	30	F	1	Psoriasis; mitochondrial dysfunction
2	28	F	2	Carnitine palmitoyl transferase deficiency
3	39	F	2	Polymyositis; carnitine palmitoyl transferase deficiency
4	50	M	2	Carnitine palmitoyl transferase deficiency
5	53	M	5	Psoriasis; APLA; mitochondrial dysfunction
6	56	F	1	Complex 2 disorder mitochondrial respiratory chain; APLA; celiac sprue
7	54	F	2	Lactate dehydrogenase deficiency
8	49	F	5	Carnitine palmitoyl transferase deficiency
9	60	F	3	Carnitine palmitoyl transferase deficiency; APLA
10	63	F	18	Mitochondrial dysfunction

Mitochondrial dysfunction was defined as elevated lactic acid while at rest without any explanation for the elevation, such as liver failure

*APLA* anti-phospholipid antibody syndrome

The clinical features that brought these patients to medical attention were abdominal pain, bloating, diarrhea and fatigue associated in many cases with joint pains involving predominantly the wrists, knees and ankles. Gastroenterologist had performed endoscopies and colonoscopies on all of these patients. One patients was diagnosed with celiac sprue, but still had ongoing symptoms after elimination of gluten. The remainder of the patients had been given the diagnosis of IBS. All of the patients had deficiencies in 25-OH Vitamin D, often with levels less than 20 ng/ml (Normal 30–100 ng/ml), suggesting some degree of malabsorption (Table 2). One patient had an episode of uveitis that was unexplained, but may have be related to gastrointestinal inflammation (Rachitskaya et al. 2010).

**Table 2:** This table summarizes the foods to which the patients had positive food reactivities based on specific IgG ImmunoCAP^®^ studies

Patient	Food hypersensitivities	Autoantibodies
1	Wheat, dairy, eggs, beef, corn	ASCA, ANA
2	Wheat, dairy, beef, corn, rice, tomato, carrot, broccoli, apple	ASCA, ANA, anti-SP1
3	Wheat, dairy	ASCA, ANA
4	Wheat, eggs, dairy, banana, beef, rice, corn, almond	Atypical pANCA, anti-CA6, anti-PSP
5	Wheat, soy, dairy, corn banana, egg, peanut, shrimp, almond, tomato	ANA, RF
6	Wheat, eggs, dairy, banana, beef, pork, corn, rice, tomato, soy	ANA, anti-gliadin, APLA
7	Rice, almonds, eggs, banana	ASCA, ANA, anti-SP1
8	Wheat, dairy, tomato, eggs	Anti-Ro
9	Wheat, eggs, dairy, soy, corn, strawberry	ASCA, ANA, anti-Ro
10	Wheat, eggs, dairy, corn, beef	Anti-SP1

Autoantibodies were determined by ELISA assays

*ASCA* anti-*Saccharomyces cerevesiae* antibodies, *anti*-*CA6* anti-carbonic anhydrase 6, *anti*-*PSP* anti-parotid secretory protein, *anti*-*SP1* anti-salivary gland protein 1

The ImmunoCAP^®^ testing for IgG reactivity to foods revealed reactivities to multiple food in all cases (Table 2). Reactivity to wheat and dairy was present in all but one patient. Other common reactivities were to eggs, beef and corn. All of the patients had autoantibodies associated with SS, ANA, RF, anti-Ro, anti-salivary gland protein 1 (SP1), anti-carbonic anhydrase 6 (CA6) and/or anti-parotid secretory protein (Kyriakidis et al. 2014; Shen et al. 2012, 2014). Most of the patients had antibodies associated with inflammation in the gastrointestinal tract, five patients had anti-*S. cerevesiae* antibodies (ASCA), one patient had atypical pANCA and one patient had anti-gliadin antibodies (Torok et al. 2004; Beniwal and Harrell 2010).

As shown in Table 3, all patients, except for two, were able to eliminate all the foods from their diets to which they had hypersensitivity. The eight patients with complete elimination diets had full resolution of abdominal pain, bloating, diarrhea and joint pain on the restricted diet. The two patients with incomplete elimination diets did eliminate wheat and dairy from their diets and noted improvement but not resolution of their abdominal symptoms. In all cases, symptoms returned when culprit foods were re-introduced. Re-introduction of food was done one food at a time. In most cases, ingestion of either wheat or dairy returned the gastrointestinal symptoms that had occurred before the elimination diet. Over time, some patients were able to re-introduce some foods without symptoms, such as tomato, pork and rice.

**Table 3:** This table summarizes the symptoms attributable to food hypersensitivities in each of the patients, their symptoms on their elimination diets and the foods added back that caused the initial symptoms to recur

Patient	Symptoms ascribed to food hypersensitivity	Symptoms remaining on food elimination	Recurrence of all symptoms with food re-challenge
1	Bloating, diarrhea, joint pain, fatigue	Mild fatigue	Yes; wheat or dairy
2	Bloating, diarrhea, joint pain, fatigue	All improved, but never complete food elimination	Yes; wheat, corn or dairy
3	Bloating, diarrhea, joint pain, fatigue	Mild fatigue	Yes; wheat or dairy
4	Bloating, diarrhea, joint pain, fatigue, scleritis	Mild fatigue	Yes; wheat, eggs, dairy or beef
5	Bloating, joint pain, fatigue	Mild fatigue	Yes; wheat or dairy
6	Bloating, diarrhea, joint pain, fatigue	Mild fatigue	Yes; dairy, corn, soy or rice; she would not try wheat
7	Bloating, diarrhea, joint pain, fatigue	Fatigue	Yes; rice, eggs or banana
8	Bloating, diarrhea, joint pain, fatigue	Mild fatigue	Yes; wheat or diary or eggs
9	Bloating, diarrhea, joint pain, fatigue	All improved, but never complete food elimination	Yes; wheat or dairy
10	Bloating, diarrhea, joint pain, fatigue	Mild fatigue	Yes; wheat, corn or dairy

While fatigue was a major component in the symptoms of all these patients, elimination diets improved but did not resolve it. Further improvement in fatigue came from addressing the underlying metabolic diseases. Patients with mitochondrial dysfunction from any cause received CoQ10, creatine, carnitine, folic acid and alpha-lipoic acid (Tarnopolsky 2008; Abdullah et al. 2012; Ambrus 2009). The patient with lactate dehydrogenase deficiency was treated with a diet avoiding complex carbohydrates and rich in simple sugars (Weinstein and Wolfsdorf 2002).

## Discussion

The current study was an observational study made on a group of patients with SS derived from a University autoimmune disease clinic that also specializes in metabolic muscle diseases. It is therefore a very selected group of patients. Nonetheless, these patients presented with symptoms that are common to SS patients everywhere and often ascribed to IBS, bloating, diarrhea, joint pain and fatigue (Bengtsson et al. 2011; Daniels et al. 2011). Patients were identified to have various food hypersensitivities by IgG reactivity to these foods. Diets eliminating these foods led to resolution of symptoms that recurred with re-introduction of the foods. Resolution of fatigue required attention to not only the food hypersensitivities but also to metabolic disorders.

The incidence of celiac sprue, or gluten hypersensitivity, is estimated to be 1 % of the population and to be present in many patients with Sjogren’s syndrome (Rashtak et al. 2009). Celiac sprue is associated with abdominal pain, diarrhea, bloating, arthritis, uveitis, fatigue, iron deficiency and various vitamin deficiencies, including vitamin D (McGough and Cummings 2005). It is known that many patients with celiac sprue to not respond to elimination of gluten alone. Many theories exist regarding the failure of gluten elimination alone to resolve symptoms in these patients (Farrell and Kelly 2002).

More recently, it has been recognized that hypersensitivity reactions can occur to food other than gluten. In fact, gastroenterologists discuss the entity of non-celiac sprue wheat hypersensitivity as a common entity (Battais et al. 2003). The idea that hypersensitivities to other foods, such as dairy, eggs and beef, giving similar symptoms to celiac sprue is a relatively new concept that is undergoing intense study (Zuo et al. 2007). Allergist studying “food allergies” recognize IgE mediated reactions to foods that cause immediate diarrhea, skin rash and swelling upon ingestion of the foods (Kurowski and Boxer 2008). It is being recognized more by gastroenterologists than allergists that hypersensitivities to foods cause delayed reactions (8–48 h after ingestion) that most commonly include bloating, diarrhea and fatigue (Jyonouchi 2008; Wolfe and Aceves 2011). It is also recognized that any type of gastrointestinal inflammation can lead to secondary arthralgia/arthritis most commonly involving the wrists, knees and ankles (Holden et al. 2003). Our patients clearly had symptoms suggestive of food hypersensitivities, with blood test demonstrating food sensitivities and resolution of symptoms with elimination diets that recurred with reintroduction of foods. These data suggest that hypersensitivity to these foods was a critical driver for these symptoms. The mechanism by which these food hypersensitivities caused these symptoms is entirely unclear, and should be the topic of future studies.

That these symptoms occurred in a population of patients with SS raises several interesting issues. First, it could be that food hypersensitivities and SS are both common entities and we happened to find patients in which they co-exist. This would be supported by the fact that many of the patients had gastrointestinal symptoms before the identification of SS. However, SS can frequently exist for several years before it is diagnosed (Akpek et al. 2015). Second, the SS patients may have been a unique subset. It is true that all the patients had autoantibodies and met ACR criteria for SS, however, many lacked anti-Ro or anti-La antibodies. Several patients had anti-SP1 antibodies. Whether anti-SP1, anti-CA6 and/or anti-PSP denote different subsets of SS is currently being evaluated in several studies (Suresh et al. 2015; Ambrus et al. 2012). Lastly, all of the patients in these groups of SS patients had underlying metabolic diseases. It is possible that the metabolic diseases participated in the development of both the food hypersensitivities and the SS. One scenario that could be envisioned, and would have to be evaluated in future studies, is that the underlying metabolic disease led to increased susceptibility to infection and increased difficulty in clearing infections. Infections in the gastrointestinal tract, with enteroviruses for example, would lead to reactivity to foods that were commonly in the diet. At the same time, infections in the salivary and lacrimal glands, with CMV or mumps for example, could initiate the events leading to SS. Many other scenarios can be envisioned. The bias of this study is that patients were selected from a clinic that specializes in autoimmune diseases and metabolic diseases. Further studies will have to evaluate these issues in a broader population of SS patients.

In summary, patients selected from an academic autoimmune disease and metabolic disease clinic identified 10 patients with SS and IBS like symptoms that were caused by hypersensitivity to foods, the most common being wheat and dairy. Fatigue in these patients was related to food hypersensitivities and underlying metabolic disorders. Treatment of all of these issues is necessary to help these patients feel better and have a better quality of life.

## Abbreviations

SS: Sjogren’s syndrome
IBS: irritable bowel syndrome
ASCA: anti-*Saccharomyces cerevesiae* antibodies
Anti-CA6: anti-carbonic anhydrase 6
Anti-PSP: anti-parotid secretory protein
Anti-SP1: anti-salivary gland protein 1
Atypical pANCA: atypical peri-nuclear anti-neutrophil cytoplasmic antibodies
APLA: anti-phospholipid antibody
